# Serum cytokine concentrations, flavonol intake and colorectal adenoma recurrence in the Polyp Prevention Trial

**DOI:** 10.1038/sj.bjc.6605915

**Published:** 2010-10-05

**Authors:** G Bobe, G Murphy, P S Albert, L B Sansbury, E Lanza, A Schatzkin, N H Colburn, A J Cross

**Affiliations:** 1 Laboratory of Cancer Prevention, Center for Cancer Research, National Cancer Institute (NCI), National Institutes of Health (NIH), Department of Health and Human Services (DHHS), Building 576, Room 101, 1050 Boyles Street, Frederick, MD 21702, USA; 2 Infection and Immunoepidemiology Branch, Division of Cancer Epidemiology and Genetics (DCEG), NCI, NIH, DHHS, 6120 Executive Boulevard, EPS 3034, Rockville, MD 20892, USA; 3 Biostatistics and Bioinformatics Branch, National Institute of Child Health and Human Development, NIH, DHHS, 6100 Executive Boulevard, Room 7B05, Rockville, MD 20892, USA; 4 Epidemiology and Genetics Research Program, Division of Cancer Control and Population Science, NCI, NIH, DHHS, 6130 Executive Boulevard, EPN 5106, Rockville, MD 20892, USA; 5 Nutritional Epidemiology Branch, DCEG, NCI, NIH, DHHS, 6120 Executive Boulevard, EPS 3050, Rockville, MD 20892, USA

**Keywords:** cancer prevention, colorectal adenoma, colorectal cancer, cytokines, flavonols

## Abstract

**Background:**

Serum cytokine concentrations may reflect inflammatory processes occurring during the development of colorectal neoplasms. Flavonols, bioactive compounds found in plant-based foods and beverages, may inhibit colorectal neoplasms partly by attenuating inflammation.

**Methods:**

Using logistic regression, we estimated odds ratios (ORs) and 95% confidence intervals (CIs) to investigate the association between serum concentrations of interleukin (IL)1*β*, 2, 8, 10, 12p70, granulocyte macrophage colony stimulating factor, interferon-*γ*, and tumour necrosis factor-*α*, measured over time, flavonol intake, estimated from a flavonol database used in conjunction with a food frequency questionnaire, and adenoma recurrence in 872 participants from the intervention arm of the Polyp Prevention Trial.

**Results:**

Decreased IL-2 concentration during the trial increased the risk of any adenoma recurrence (4th *vs* 1st quartile, OR=1.68, 95% CI=1.13–2.49), whereas decreased IL-1*β* or IL-10 reduced the risk of advanced adenoma recurrence (OR=0.37, 95% CI=0.15–0.94; OR=0.39, 95% CI=0.15–0.98, respectively). Individuals with flavonol intake above the median (29.7 mg per day) and decreased cytokine concentrations had the lowest risk of advanced adenoma recurrence.

**Conclusion:**

Overall, no consistent associations were observed between serum cytokine profile and colorectal adenoma recurrence; however, decreased cytokine concentrations during high flavonol consumption may indicate prevention of colorectal neoplasms.

Growing evidence suggests that inflammation is important in carcinogenesis, including colorectal cancer (Lin and Karin, 2007). Cytokine concentrations in either serum or tumours may be useful indicators of inflammation and risk of neoplastic changes (Pellegrini *et al*, 2006; Cui and Florholmen, 2008). Compared with healthy individuals, serum cytokine concentrations of interleukin-2 (IL-2) are reported to be lower, whereas concentrations of IL-8, IL-10, IL-12, granulocyte macrophage colony stimulating factor (GMCSF), interferon (IFN)-*γ*, and tumour necrosis factor (TNF)*α* are higher in individuals with colorectal adenomas in some studies (Berghella *et al*, 1997; Mroczko *et al*, 2001; Galizia *et al*, 2002; Ordemann *et al*, 2002; Contasta *et al*, 2003; Roselli *et al*, 2003; Kaminska *et al*, 2005; Kim *et al*, 2008).

Flavonols are a flavonoid subgroup of bioactive polyphenols that are present in many plant-based foods and beverages (Chun *et al*, 2007; Bobe *et al*, 2008). The literature and our own studies suggest that flavonols are one of the flavonoid subgroups most effective in decreasing the risk of advanced and high-risk colorectal adenoma recurrence (Bobe *et al*, 2008, 2010) and colorectal cancer (Rossi *et al*, 2006; Theodoratou *et al*, 2007). Several human studies indicate that flavonols have anti-inflammatory properties (Chun *et al*, 2008; Boots *et al*, 2009; Bobe *et al*, 2010), which may be one of the several molecular mechanisms by which flavonols may inhibit the growth of colorectal neoplasms. The aims of this study were to examine whether serum concentrations of IL-1*β*, IL-2, IL-8, IL-10, IL-12p70, GMCSF, IFN*γ*, and TNF*α* were associated with flavonol intake or could predict colorectal adenoma recurrence. In addition, we investigated whether a predicted protective effect of flavonol intake might be mediated by changes in serum cytokine concentrations.

## Materials and methods

### Study design and outcome

The Polyp Prevention Trial (PPT) was a 4-year multi-centre, randomised, nutritional intervention trial to evaluate whether colorectal adenoma recurrence can be inhibited by increasing fibre, fruit, and vegetable consumption and decreasing the proportion of fat in the diet. The study has previously been described in detail (Schatzkin *et al*, 2000; Lanza *et al*, 2001). The main requirement was that study participants had at least one histologically confirmed colorectal adenoma identified by complete colonoscopy in the 6 months before study entry. Of the 1905 participants who completed the trial by undergoing a colonoscopy at the end of year 4, 958 were in the intervention arm. Our study included the 872 participants in the intervention arm with available dietary data for any of the first 3 years of the trial and serum from baseline (T0) and either from year 1 (T1) or 3 (T3). Two pathologists independently examined all lesions for histological features and degree of atypia. Adenoma recurrence was defined as: any (⩾1 adenoma, *n*=348), high risk (⩾3 adenomas or ⩾1 advanced adenoma, *n*=100), or advanced (⩾1 adenoma of ⩾1 cm in size, having ⩾25% villous component, or exhibiting high-grade dysplasia, *n*=49). The institutional review boards of the National Cancer Institute and each participating centre approved the study, and all participants provided written informed consent.

### Lifestyle and flavonol data

At T0 and at each of the annual follow-up visits (T1, T2, T3, and T4), participants were asked to complete an interviewer-administered questionnaire about demographics, family history, and use of medication or supplements (including name and dosage), as well as a self-administered food frequency questionnaire (FFQ) that was reviewed with a certified nutritionist. The FFQ was specifically designed and validated to accurately measure fat, fibre, fruit, and vegetable consumption (Block *et al*, 1990). Relative to 24 h dietary recall and 4-day food record data, the FFQ slightly overestimated fat and underestimated fibre, fruit and vegetable intake, and had acceptable correlations of macronutrients and micronutrients (Caan *et al*, 1999; Lanza *et al*, 2001). The average flavonol intake for the first 3 years of the trial was estimated using 55 of the 119 questions on the FFQ using the 2007 flavonoid database (U.S. Department of Agriculture, 2007) and was calculated as the sum of isorhamnetin, kaempferol, myricetin, and quercetin.

### Serum data

At each annual visit, participants provided an overnight fasting blood sample, the serum from which was stored at −70°C until analysis. Among the 872 participants, 23 and 69 had no available samples at T1 and T3, respectively. Serum concentrations of IL-1*β*, IL-2, IL-8, IL-10, IL-12p70, GMCSF, IFN*γ*, and TNF*α* were measured at T0, T1, and T3 by the Clinical Support Laboratory of SAIC Frederick, Inc. (Frederick, MD, USA) using a commercially available multiplex 96-well enzyme-linked immunoabsorbent assay kit (MS6000 Human Pro-Inflammatory 9-Plex Ultra-Sensitive Kit K11007; Meso Scale Diagnostics, Gaithersburg, MD, USA) on a Sector Imager 6000 according to the manufacturer's recommendation (Meso Scale Diagnostics). Study samples were run with two pooled serum samples and three assay specific standards in duplicate and the average of the duplicate was used. Fewer than 1% of the samples were below the detection limit, and the interassay coefficient of variation (CV) was below 15%.

### Statistical analyses

Statistical analyses were performed using SAS, version 9.1 (SAS, Inc., Cary, NC, USA) software. Baseline characteristics, average dietary intake for the first 3 years of the trial, and serum cytokine concentrations were evaluated by adenoma recurrence at T4 (no *vs* any, high-risk, or advanced adenoma recurrence) using Wilcoxon rank-sum test for continuous variables and Fisher's exact test for categorical variables and are shown as medians and interquartile ranges (IQRs). Spearman's correlation coefficients between serum cytokine concentrations were calculated. The association between serum cytokine concentrations and flavonol consumption during the first 3 years of the trial was evaluated with the Kruskal–Wallis test and multiple linear regression models.

We defined trial cytokine concentrations as the geometric mean of T1 and T3. Cytokine concentration changes during the trial were defined as the geometric mean of T1 and T3 minus the baseline values. The association between cytokine changes and colorectal adenoma recurrence was estimated by odds ratios (ORs) and 95% confidence intervals (CIs) using logistic regression. A trend test was performed using the median values of each quartile as a continuous variable in a logistic regression model. The median values of both flavonol intake and cytokine changes were used as cutoffs (⩽median, >median) to examine the combined effect of flavonol intake and cytokine changes on colorectal adenoma recurrence. Potential confounders (listed in Table 1) were added to the models in a stepwise manner and remained in the model if they changed the association by >10%, were associated with both study variables, and had a *χ*^2^ *P*-value ⩽0.20. All *P*-values corresponded to two-sided tests and were considered to be significant when *P*⩽0.05.

## Results

At the end of the 4-year trial, 40% of participants had at least 1 adenoma, 11% had high-risk adenoma, and 6% had an advanced adenoma recurrence (Table 1). Compared with baseline, flavonol consumption increased two-fold from 14.6 to 29.7 mg per day during the first 3 years of the trial (Bobe *et al*, 2010). Adenoma recurrence was more common in men, older individuals, and individuals that ate a greater percentage of calories from fat during the first 3 years of the trial, and less common in women who used hormone therapy. Individuals who had recurrence of a high-risk or advanced adenoma consumed less fibre (limited to individuals with a high-risk adenoma), fruits and vegetables, flavonols, and dry beans (Table 1). Serum concentrations of IL-1*β*, IL-2, IL-8, IL-10, GMCSF, IFN*γ*, and TNF*α*, either at baseline, during the first 3 years of the trial, or from baseline to during the trial, were not associated with colorectal adenoma recurrence; with the exception that IL-12p70 was lower at baseline in individuals with high-risk and advanced adenoma recurrence than in individuals with no adenoma recurrence (Table 1; data not shown). Of the eight serum cytokines measured, only IFN*γ* concentrations differed across quartiles of flavonol intake; individuals in the lowest flavonol intake quartile had higher IFN*γ* concentrations compared with individuals in the higher 3 flavonol intake quartiles (Table 2).

No statistically significant associations were observed between serum cytokine concentrations during the trial (defined as the mean of concentration at T1 and T3) and adenoma recurrence (data not shown). In contrast, a decrease in IL-2 concentrations during the trial (the mean trial level minus the baseline concentration) was associated with increased risk of any adenoma recurrence (lowest *vs* highest quartile of change in cytokine concentration: OR=1.68, 95% CI=1.13–2.49), whereas a decrease in IL-1*β* or IL-10 reduced the risk of advanced adenoma recurrence (OR=0.37, 95% CI=0.15–0.94 and OR=0.39, 95% CI=0.15–0.98, respectively; Table 3).

**Figure 1:**
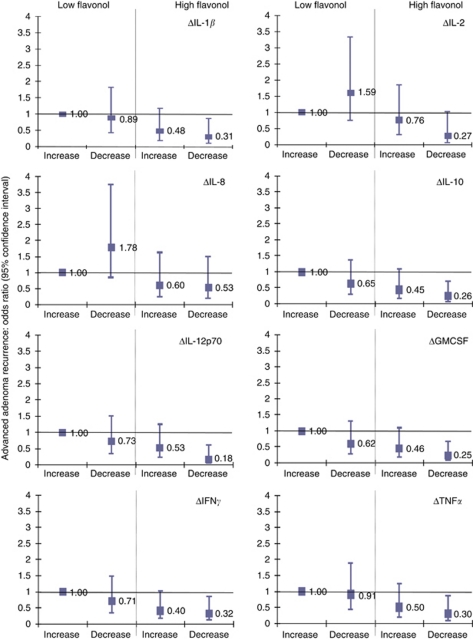
Association between the combination of high (>29.7 mg per day) or low (⩽29.7 mg per day) flavonol intake during the trial and change in serum concentration of cytokines (defined as the geometric mean of T1 and T3 minus baseline values) on advanced colorectal adenoma recurrence among participants in the intervention arm of the Polyp Prevention Trial. The cutoff values for an increase or decrease in serum cytokine concentrations are as follows (in pg ml^−1^): ΔIL-1*β*: >0.01 (increase), ⩽0.01 (decrease); ΔIL-2: >0.06 (increase), ⩽0.06 (decrease); ΔIL-8: >0.24 (increase), ⩽0.24 (decrease); ΔIL-10: >−0.04 (increase), ⩽−0.04 (decrease); ΔIL-12p70: >−0.09 (increase), ⩽−0.09 (decrease); ΔGMCSF: >0.00 (increase), ⩽0.00 (decrease); ΔIFN*γ*: >0.07 (increase), ⩽0.07 (decrease); TNF*α*: >0.02 (increase), ⩽0.02 (decrease). The reference group is the combination of low flavonol intake and increase in cytokine concentrations.

Individuals with above median flavonol intake and equal or below median change in serum cytokines concentrations had the lowest risk of advanced adenoma recurrence for all cytokines investigated but not all were statistically significant (Figure 1). Compared with individuals with equal or below median flavonol intake and above median serum cytokine concentrations, the risk reduction was statistically significant for changes in concentrations of IL-1*β*, IL-10, IL-12p70, GMCSF, IFN*γ*, or TNF*α* (Figure 1; Supplementary Table S1). Similar results were observed for the combined effect of flavonol intake and serum cytokine concentrations at baseline (less significant effect) or during the trial (Supplementary Tables S2 and S3).

## Discussion

Previously, we reported that serum concentrations of IL-6 may be a potential risk indicator for advanced and high-risk adenoma recurrence; furthermore, dietary flavonols decrease elevated IL-6 concentrations and decrease the risk of advanced and high-risk adenoma recurrence (Bobe *et al*, 2010). In the current study, we examined serum concentrations of eight cytokines (IL-1*β*, IL-2, IL-8, IL-10, IL-12p70, GMCSF, IFN*γ*, and TNF*α*) in relation to flavonol intake and colorectal adenoma recurrence and found none to be associated with flavonol intake and with colorectal adenoma recurrence. Only IFN*γ* concentrations varied significantly across flavonol intake quartiles. Serum cytokine concentrations were not associated with colorectal adenoma recurrence with the exception that a decrease in IL-2 concentrations during the trial increased the risk of any adenoma recurrence, and a decrease in IL-1*β* or IL-10 reduced the risk of advanced adenoma recurrence. Individuals with high flavonol intake (above 29.7 mg per day) and a decrease in serum concentrations of six of the eight measured cytokines had the lowest risk of advanced adenoma recurrence. Thus, our results suggest that there is not a consistent association between serum cytokine profile and colorectal adenoma recurrence; however, a decrease in cytokine concentrations during high flavonol consumption (>29.7 mg per day) may indicate a lower risk for advanced colorectal adenoma.

Chronic inflammation, involving many pro- as well as anti-inflammatory cytokines, is one of the many mechanisms reported to promote colorectal carcinogenesis (Lin and Karin, 2007). Similar to our findings, lower serum concentrations of IL-2 have been reported in colorectal adenoma and cancer patients *vs* healthy individuals (Berghella *et al*, 1997; Contasta *et al*, 2003). IL-2 is a lymphokine that enhances the growth and cytotoxic response of activated T cells and is used as an adjuvant treatment of solid tumours (Grande *et al*, 2006). We anticipated an increase in serum IL-1*β* in individuals with advanced adenoma recurrence, although Roselli *et al* (2003) did not observe differences in serum IL-1*β* concentrations between healthy and colorectal adenoma or cancer patients, because IL-1*β* initiates the pro-inflammatory cascade and is necessary for tumour invasion and metastasis (Apte *et al*, 2006; Krelin *et al*, 2007). Furthermore, the gene and protein expression of IL-1*β* is higher in colorectal adenoma and adenocarcinoma relative to normal colon tissue (Miki *et al*, 2002; Schetter *et al*, 2009). The role of IL-10 in colorectal carcinogenesis is complex as it can alternately promote and inhibit carcinogenesis (Moore *et al*, 2001; Lin and Karin, 2007; Uronis *et al*, 2009), and both increased and decreased IL-10 gene or protein expression have been found in tumour tissue (Miki *et al*, 2002; Csiszar *et al*, 2004; Schetter *et al*, 2009; Stanilov *et al*, 2009). Compared with healthy individuals, colorectal cancer patients have significantly higher IL-10 concentrations but individuals with adenomas do not (Berghella *et al*, 1997; Ordemann *et al*, 2002; Contasta *et al*, 2003; Stanilov *et al*, 2009), suggesting that IL-10 may be a better risk indicator for more advanced tumour stages.

Although we did not find associations for IL-8, IL-12p70, GMCSF, IFN*γ*, and TNF*α* and adenoma recurrence, they may serve as risk indicators for more advanced tumour stages. Elevated concentrations of IL-8, TNF*α*, IL-12 (a heterodimer consisting of IL-12p40 and IL-12p70), GMCSF, and IFN*γ* in blood have been reported in colorectal cancer patients in some but not all studies (Berghella *et al*, 1996, 1997, 2002; Mroczko *et al*, 2001, 2007; Contasta *et al*, 2003; Roselli *et al*, 2003; Kaminska *et al*, 2005; Schetter *et al*, 2009; Stanilov *et al*, 2009). Low baseline values, large CVs, and a limited dynamic range in most human samples combined with smaller increases in cytokine concentrations in blood in early *vs* later stages of colorectal neoplasia may limit the potential of IL-8, TNF*α*, IL-12p70, GMCSF, and IFN*γ* as risk indicators for colorectal cancer prevention, although higher concentrations of TNF*α* in blood have been observed in colorectal adenoma patients compared with healthy individuals (Berghella *et al*, 1996; Roselli *et al*, 2003; Kim *et al*, 2008).

Flavonols are naturally occurring bioactive polyphenols found in various plant-based foods and beverages, especially in apples, beans, onions, and tea (Chun *et al*, 2007; Bobe *et al*, 2008), that may attenuate secretion of pro-inflammatory cytokines in humans (Chun *et al*, 2008; Boots *et al*, 2009; Egert *et al*, 2010). There are multiple molecular mechanisms by which flavonols may attenuate inflammatory processes, including inhibiting the activity of dendritic and mast cells (Park *et al*, 2008; Huang *et al*, 2010), attenuating nitric oxide production (Wang *et al*, 2006) and pathways induced by cyclooxygenase and lipoxygenase (Wang *et al*, 2006; Bednar *et al*, 2007; Lee *et al*, 2010a), inducing the expression of non-steroidal anti-inflammatory drug activated gene-1 (Lim *et al*, 2007), and decreasing the activity of phopholipase A2 (Moon *et al*, 2008), peroxisome proliferator activated receptor *γ* (Lian *et al*, 2008), and nuclear factor *κ*B (Ruiz and Haller, 2006; Park *et al*, 2008). In the United States, the median flavonol intake is estimated to be ∼10–12 mg per day, with a range between 0 and 40 mg, the primary dietary flavonols being quercetin (70% of total flavonols), kaempferol (16%), myricetin (12%), and isorhamnetin (2%) (Peterson JJ, personal communication). Previously, we reported that high flavonol intake (>30 mg per day) may decrease serum IL-6 and the incidence of high-risk and advanced adenoma recurrence in the PPT (Bobe *et al*, 2010). In the current study, we observed that high flavonol consumption (>21 mg per day) may also decrease serum IFN *γ*. Flavonol supplementation studies do not usually observe changes in blood cytokines except in individuals with elevated baseline values combined with an inflammatory challenge (Nieman *et al*, 2007; Boots *et al*, 2008, 2009). Studies in cell culture (Nair *et al*, 2002; Min *et al*, 2007; Bandyopadhyay *et al*, 2008; Okoko and Oruambo, 2009) and animal models (Kwon *et al*, 2005; Camuesco *et al*, 2006) often use an inflammatory challenge to measure the flavonol-induced attenuation of cytokine secretion and gene expression. Thus, flavonols may primarily benefit individuals at increased inflammation risk, or flavonol-induced changes in inflammatory markers may be too small to be detected when cytokine concentrations are within the normal dynamic range.

We observed the lowest risk for advanced adenoma recurrence with high flavonol intake (>30 mg per day) and a concurrent decrease in serum cytokine concentrations. In addition to their anti-inflammatory properties, dietary flavonols are thought to inhibit carcinogenesis through several other pathways. Flavonols can decrease various forms of DNA damage (Duthie and Dobson, 1999; Wilms *et al*, 2005); they have anti-mutagenic properties (Ruf *et al*, 2003; Gupta *et al*, 2010), stabilise the helical structure of DNA (Kanakis *et al*, 2007), and enhance DNA repair (Min and Ebeler, 2009). Furthermore, flavonols can scavenge reactive oxygen species (Kim *et al*, 2006; Wang *et al*, 2006), bind metals (Guo *et al*, 2007), decrease lipid peroxidation (Lee *et al*, 2010c), inhibit the activity of phase I procarcinogen activating enzymes (Si *et al*, 2009; Lam *et al*, 2010; Tiong *et al*, 2010), and induce the expression of phase II carcinogen detoxification enzymes (Lam *et al*, 2010) and antioxidant proteins (Kimura *et al*, 2009). In the tumour promotion and progression stage, flavonols inhibit transformation of pre-carcinogenic cells (Ichimatsu *et al*, 2007; Lee *et al*, 2008) and proliferation of cancer cells (Richter *et al*, 1999; Kim *et al*, 2005) by inducing cell cycle arrest and apoptosis (Choi *et al*, 2008; Jeong *et al*, 2009). Furthermore, flavonols inhibit tumour angiogenesis and invasiveness by repressing expression of the angiogenesis-promoting vascular endothelial growth factors (Kim *et al*, 2006; Luo *et al*, 2009; Lee *et al*, 2010b) and invasion-promoting matrix metalloproteinases, respectively (Vijayababu *et al*, 2006; Lin *et al*, 2008; Phromnoi *et al*, 2009; Zhang and Zhang, 2009). Thus, the decrease in cytokine concentrations may be, at least in part, a result of flavonols inhibiting adenoma progression rather than a direct effect on cytokine expression and secretion.

One of the strengths of this study is the detailed end point information, which included complete colonoscopies and histologic characterisation of all lesions by two pathologists, decreasing the risk of misclassification. A second strength is the prospective and repeated collection of dietary exposure. The modified FFQ used in the PPT was specifically developed to accurately measure high fruit and vegetable consumption (Block *et al*, 1990; Lanza *et al*, 2001) and was linked to the recently released validated USDA flavonoid database (U.S. Department of Agriculture, 2007). The accuracy of the FFQ was further improved as registered dieticians reviewed the FFQ with participants (Caan *et al*, 1999). A third strength is the repeated collection of serum, which allowed us to look at changes during, what may be, early stages of colorectal carcinogenesis.

Limitations of the study include the fact that the PPT is a study of individuals with a history of adenomas, most of whom were Caucasians already engaged in a health-promoting lifestyle. Random as well as systematic measurement error related to the dietary assessment, the flavonoid database, and the participants’ knowledge of the expected dietary patterns may be present and could bias risk estimates. The low abundance, high CVs, daily fluctuations, short half-lives, lack of specificity for location, strength and type of inflammation, and the limited dynamic ranges of cytokines in most human serum samples could partly explain the inconsistent results for serum cytokines as markers of colorectal neoplasia and limit the usefulness of many cytokines as biomarkers. Observed differences may have arisen by chance as participants were not randomly assigned to a specific flavonol diet, the number of cases of advanced adenoma recurrence was small, and multiple cytokines were tested for multiple outcomes (multiple testing). However, the consistent lower risk of advanced adenoma recurrence with decreasing cytokine concentration during high flavonol consumption is unlikely due to chance. Besides flavonols, other flavonoid subgroups, such as anthocyanins, flavan-3-ols, flavones, and isoflavonoids, have cancer-protective and anti-inflammatory properties (Yoon and Baek, 2005; Ferguson and Philpott, 2007; Wang and Stoner, 2008). We focused on flavonols because they were the flavonoid subgroup most protective against advanced adenoma recurrence in the PPT (Bobe *et al*, 2008); the intake ranges of other flavonoid subgroups in the PPT may be too limited to detect associations.

In conclusion, our results suggest that a decrease in cytokine concentrations during high flavonol consumption may serve as a risk indicator for colorectal cancer prevention. Verification of these results in other prospective cohorts with high quality and repeated dietary and serum cytokine measures is needed to clarify the role of serum cytokines as indicators of a chemopreventive response to dietary flavonols.

## Figures and Tables

**Table 1:** Proportions and medians (IQRs) of participant characteristics in the intervention arm of the Polyp Prevention Trial by adenoma recurrence at T4 (*n*=872)

	**Adenoma recurrence (T4)**						
	**None**	**Any**		**High risk**		**Advanced**	
**Characteristics**	**Median (IQR) or %**	**Median (IQR) or %**	***P*-value^a^**	**Median (IQR) or %**	***P*-value^a^**	**Median (IQR) or %**	***P*-value^a^**
Sample size (%)	524 (60)	348 (40)		100 (11)		49 (6)	
							
*Baseline (T0)*							
Gender (% male)	64	70	0.05	76	0.02	69	0.53
Race (% Caucasian)	88	90	0.58	90	0.73	86	0.64
Education (% ⩽high school)	22	27	0.11	31	0.07	31	0.21
Family history of colorectal cancer (% yes)^b^	27	26	0.88	30	0.46	27	1.00
Smoker (% current)	11	14	0.21	18	0.07	14	0.48
NSAID use (% yes)^c^	35	37	0.67	33	0.65	31	0.54
Supplement use (% yes)^c^	45	43	0.68	38	0.23	37	0.30
Hormone therapy (% yes)^c^	13	9	0.05	6	0.04	8	0.38
Age (years)	60.0 (52.0–67.0)	64.0 (57.0–70.0)	<0.0001	66.0 (58.0–71.0)	<0.0001	66.0 (60.0–71.0)	0.0006
Body mass index (kg m^−2^)	27.5 (24.8–30.3)	27.6 (25.1–30.6)	0.36	28.3 (25.4–31.1)	0.06	28.6 (26.1–32.4)	0.01
Physical activity (hours per week)^d^	8.50 (4.00–15.1)	8.46 (3.82–16.0)	0.93	8.27 (3.33–13.1)	0.21	7.16 (2.67–12.0)	0.19
							
*Serum cytokines (pg ml* ^−*1*^ *)*							
Interleukin-1*β*	0.36 (0.22–0.60)	0.37 (0.22–0.63)	0.88	0.37 (0.21–0.72)	0.95	0.34 (0.19–0.62)	0.42
Interleukin-2	0.72 (0.34–1.45)	0.69 (0.37–1.60)	0.61	0.66 (0.42–1.54)	0.50	0.83 (0.48–1.60)	0.26
Interleukin-8	10.4 (7.81–13.7)	10.2 (8.09–14.1)	0.58	10.8 (8.47–16.3)	0.14	11.0 (9.05–15.2)	0.19
Interleukin-10	3.27 (2.16–5.81)	3.22 (2.07–6.07)	0.48	3.23 (2.14–4.49)	0.26	2.80 (2.03–4.00)	0.08
Interleukin-12p70	3.16 (1.69–7.69)	2.99 (1.47–7.16)	0.35	2.61 (1.36–5.64)	0.04	2.22 (1.31–4.84)	0.05
GMCSF	0.83 (0.41–1.90)	0.70 (0.36–1.89)	0.40	0.61 (0.33–1.44)	0.08	0.61 (0.36–1.07)	0.12
Interferon-*γ*	1.29 (0.85–2.37)	1.33 (0.83–2.28)	0.91	1.29 (0.87–2.39)	0.60	1.23 (0.86–1.84)	0.87
Tumour necrosis factor *α*	8.36 (6.80–10.1)	8.22 (6.79–10.3)	0.77	8.57 (7.00–10.6)	0.09	8.28 (6.76–10.0)	0.97
							
*Trial* (*T1,2,3*)^e^							
*Dietary intake*							
Alcohol (g per day)	0.90 (0.00–8.76)	0.96 (0.00–8.26)	0.51	0.98 (0.00–5.63)	0.42	0.98 (0.00–4.93)	0.24
Energy (1000 kcal per day)	1.78 (1.52–2.09)	1.79 (1.54–2.07)	1.00	1.80 (1.56–1.99)	0.92	1.82 (1.60–1.99)	0.95
Fat (% kcal per day)	22.4 (18.5–26.6)	22.9 (19.7–28.1)	0.03	25.1 (20.9–30.0)	0.0003	27.4 (22.2–30.6)	0.0002
Fibre (g per day)	32.1 (24.0–40.8)	30.9 (22.8–39.0)	0.13	29.4 (21.6–36.2)	0.01	29.4 (21.5–37.7)	0.08
Fruits and vegetables (servings per day)	5.72 (4.43–7.15)	5.65 (4.48–6.99)	0.54	5.24 (4.36–6.56)	0.03	4.98 (4.06–6.03)	0.01
Flavonols (mg per day)	29.7 (21.4–40.8)	29.7 (21.0–38.9)	0.59	25.4 (16.2–36.1)	0.005	21.0 (15.0–30.1)	0.0002
Dry beans (g per day)	31.2 (15.3–54.6)	30.3 (14.4–49.5)	0.26	23.0 (8.79–42.1)	0.005	14.0 (7.37–35.2)	0.0001
							
*Serum cytokines (pg ml*^−*1*^*)*							
Interleukin-1*β*	0.37 (0.24–0.62)	0.36 (0.23–0.59)	0.43	0.41 (0.26–0.68)	0.28	0.43 (0.25–0.68)	0.36
Interleukin-2	0.81 (0.37–1.58)	0.75 (0.35–1.41)	0.30	0.85 (0.45–1.58)	0.83	0.86 (0.51–1.65)	0.49
Interleukin-8	10.6 (8.15–15.5)	10.9 (8.36–14.7)	0.89	11.0 (8.52–16.1)	0.54	11.0 (8.98–15.2)	0.45
Interleukin-10	3.20 (2.22–5.66)	3.14 (2.10–5.56)	0.32	3.23 (2.01–4.91)	0.38	3.08 (1.87–5.66)	0.39
Interleukin-12p70	3.13 (1.66–7.09)	2.81 (1.48–6.92)	0.16	2.61 (1.47–5.29)	0.04	2.28 (1.46–5.27)	0.06
GMCSF	0.83 (0.43–1.76)	0.74 (0.36–1.81)	0.20	0.56 (0.34–1.42)	0.06	0.57 (0.35–1.32)	0.22
Interferon-*γ*	1.39 (0.93–2.32)	1.37 (0.91–2.01)	0.36	1.48 (1.00–2.41)	0.39	1.55 (1.18–2.00)	0.23
Tumour necrosis factor *α*	8.15 (6.88–9.78)	8.22 (6.99–9.98)	0.52	8.28 (7.27–11.0)	0.05	8.22 (7.00–10.0)	0.58

Abbreviations: GMCSF=granulocyte macrophage colony stimulating factor; IQR=interquartile range; NSAID=non-steroidal anti-inflammatory drug.

a All comparisons against the no adenoma recurrence group. *P-*values for differences in proportions were calculated using Fisher's exact test. *P-*values for differences in medians were calculated using Wilcoxon rank-sum test.

b Family history of colorectal cancer was defined as having ⩾1 first-degree relative with colorectal cancer at baseline.

c Regular dietary supplement use was defined as taking supplement ⩾1 weekly. Regular medication use, including NSAIDs, was defined as taking medication ⩾1 monthly. Hormone replacement therapy included both unopposed estrogen and estrogen/progestin combinations.

d Physical activity was defined as self-reported time typically spent for any type of moderate or vigorous physical activity.

e T1,2,3: mean values of the first 3 years of the trial for dietary variables and geometric mean of years 1 and 3 cytokine values.

**Table 2:** Medians (IQRs) of serum cytokine concentrations by flavonol intake during the trial (*n*=872)

	**Flavonol intake quartiles (mg per day)^b^**					
**Cytokine^a^**	**Q1: <21.1**	**Q2: 21.1–29.6**	**Q3: 29.7–40.0**	**Q4: >40.0**		
**(pg ml^−1^)**	**Median (IQR)**	**Median (IQR)**	**Median (IQR)**	**Median (IQR)**	***P* non-param.^c^**	***P* for trend^d^**
Sample size	218	218	218	218		
Interleukin-1*β*	0.41 (0.26–0.70)	0.36 (0.23–0.61)	0.35 (0.23–0.51)	0.35 (0.22–0.62)	0.14	0.09
Interleukin-2	0.80 (0.38–1.65)	0.74 (0.30–1.41)	0.780 (0.39–1.26)	0.77 (0.37–1.66)	0.69	0.28
Interleukin-8	10.8 (8.42–14.7)	11.0 (8.72–16.0)	10.5 (7.85–15.0)	10.5 (7.95–15.2)	0.62	0.90
Interleukin-10	3.41 (2.26–6.11)	3.02 (2.07–4.96)	3.15 (2.15–6.40)	3.27 (2.19–5.58)	0.53	0.81
Interleukin-12p70	3.17 (1.70–7.85)	2.73 (1.48–5.53)	3.06 (1.70–7.25)	3.02 (1.48–7.84)	0.15	0.92
GMCSF	0.82 (0.43–1.78)	0.70 (0.38–1.48)	0.82 (0.38–2.34)	0.83 (0.41–2.05)	0.35	0.74
Interferon-*γ*	1.61 (1.13–2.64)	1.20 (0.78–2.06)	1.37 (0.91–2.15)	1.28 (0.89–1.98)	0.0003	0.03
Tumour necrosis factor *α*	8.06 (6.68–10.2)	8.52 (7.05–10.2)	8.02 (6.93–9.71)	8.15 (6.95–9.65)	0.46	0.90

Abbreviations: GMCSF=granulocyte macrophage colony stimulating factor; IQR=interquartile range.

a Geometric mean of years 1 and 3 cytokine values (Trial (T1,3)).

b Participants were grouped in quartiles (Q1–Q4) by mean flavonol intake during the first 3 trial years.

c *P-*values for differences in medians among the flavonol intake quartiles were calculated based on the Kruskal–Wallis test.

d Median concentrations of each flavonol quartile were used to determine *P* for trend of the cytokine concentrations using a multiple regression model adjusting for age tertiles (<58, 58–66, >66 years), sex, average BMI (<25, 25.0–29.9, ⩾30 kg m^−2^), smoking status, and average energy intake (continuous) during the first 3 trial years. Individuals in the lowest flavonol intake quartile had higher interferon-*γ* concentrations than individuals in the three higher flavonol intake quartiles, while not differing among each other.

**Table 3:** Association between quartiles of change^a^ in serum cytokine concentrations from baseline to the levels measured during the trial (mean of T1 and T3) and colorectal adenoma recurrence in the intervention arm of the Polyp Prevention Trial (*n*=872)

	**Adenoma recurrence (T4)**						
**Cytokine**	**None**	**Any**		**High risk**		**Advanced**	
**(pg ml^−1^)**	***n* (%)**	***n* (%)**	**OR (95% CI)^b^**	***n* (%)**	**OR (95% CI)^b^**	***n* (%)**	**OR (95% CI)^b^**
*Interleukin-1β*							
Q1:>0.16	136 (62.7)	81 (37.3)	1.00	29 (13.4)	1.00	18 (8.3)	1.00
Q2: 0.02–0.16	133 (61.0)	85 (39.0)	1.05 (0.71–1.56)	22 (10.1)	0.71 (0.38–1.32)	9 (4.1)	0.48 (0.20–1.13)
Q3: −0.13–0.01	128 (58.7)	90 (41.3)	1.15 (0.78–1.71)	31 (14.2)	1.07 (0.59–1.92)	15 (6.9)	0.82 (0.39–1.76)
Q4:<−0.13	127 (58.0)	92 (42.0)	1.17 (0.79–1.73)	18 (8.2)	0.57 (0.30–1.11)	7 (3.2)	0.37 (0.15–0.94)
*P* for trend^c^			0.40		0.18		0.06
*Interleukin-2*							
Q1:>0.41	142 (65.4)	75 (34.6)	1.00	25 (11.5)	1.00	13 (6.0)	1.00
Q2: 0.07–0.41	132 (60.6)	86 (39.4)	1.22 (0.82–1.82)	21 (9.6)	0.87 (0.46–1.67)	11 (5.0)	0.90 (0.38–2.12)
Q3: −0.30–0.06	133 (61.0)	85 (39.0)	1.17 (0.79–1.74)	26 (11.9)	1.05 (0.56–1.94)	11 (5.0)	0.88 (0.37–2.07)
Q4:<−0.30	117 (53.4)	102 (46.6)	1.68 (1.13–2.49)	28 (12.8)	1.33 (0.72–2.45)	14 (6.4)	1.31 (0.58–2.95)
*P* for trend^c^			0.01		0.31		0.52
*Interleukin-8*							
Q1:>2.74	137 (63.1)	80 (36.9)	1.00	21 (9.7)	1.00	8 (3.7)	1.00
Q2: 0.25–2.74	128 (58.7)	90 (41.3)	1.19 (0.80–1.76)	20 (9.2)	0.97 (0.49–1.90)	12 (5.5)	1.55 (0.60–3.99)
Q3: −2.02–0.24	124 (56.9)	94 (43.1)	1.30 (0.88–1.93)	30 (13.8)	1.53 (0.81–2.87)	15 (6.9)	2.06 (0.83–5.13)
Q4:<−2.02	135 (61.6)	84 (38.4)	1.00 (0.67–1.48)	29 (13.2)	1.31 (0.70–2.45)	14 (6.4)	1.65 (0.66–4.14)
*P* for trend^c^			0.83		0.27		0.24
*Interleukin-10*							
Q1:>0.57	135 (62.2)	82 (37.8)	1.00	27 (12.4	1.00	18 (8.3)	1.00
Q2: −0.03–0.57	127 (58.3)	91 (41.7)	1.11 (0.75–1.65)	26 (11.9)	0.95 (0.51–1.75)	12 (5.5)	0.66 (0.30–1.47)
Q3: −0.88 to −0.04	133 (61.0)	85 (39.0)	1.02 (0.69–1.52)	24 (11.0)	0.84 (0.45–1.56)	12 (5.5)	0.61 (0.28–1.36)
Q4:<−0.88	129 (58.9)	90 (41.1)	1.16 (0.78–1.72)	23 (10.5)	0.86 (0.46–1.60)	7 (3.2)	0.39 (0.15–0.98)
*P* for trend^c^			0.50		0.62		0.04
*Interleukin-12p70*							
Q1:>0.68	137 (63.1)	80 (36.9)	1.00	24 (11.1)	1.00	15 (6.9)	1.00
Q2: −0.08–0.68	126 (57.8)	92 (42.2)	1.17 (0.79–1.73)	32 (14.7)	1.27 (0.69–2.34)	16 (7.3)	1.04 (0.48–2.26)
Q3: −1.03 to −0.09	128 (58.7)	90 (41.3)	1.15 (0.78–1.70)	24 (11.0)	0.98 (0.52–1.86)	9 (4.1)	0.63 (0.26–1.54)
Q4:<−1.03	133 (60.7)	86 (39.3)	1.10 (0.74–1.63)	20 (9.1)	0.84 (0.43–1.63)	9 (4.1)	0.60 (0.25–1.45)
*P* for trend^c^			0.78		0.43		0.21
*Granulocyte macrophage colony stimulating factor*							
Q1:>0.22	137 (63.1)	80 (36.9)	1.00	25 (11.5)	1.00	16 (7.4)	1.00
Q2: 0.01–0.22	122 (56.0)	96 (44.0)	1.31 (0.89–1.94)	27 (12.4)	1.15 (0.62–2.14)	15 (6.9)	1.02 (0.47–2.21)
Q3: −0.29–0.00	134 (61.5)	84 (38.5)	1.04 (0.70–1.55)	30 (13.8)	1.15 (0.62–2.10)	12 (5.5)	0.72 (0.32–1.62)
Q4:<−0.29	131 (59.8)	88 (40.2)	1.20 (0.81–1.78)	18 (8.2)	0.78 (0.40–1.54)	6 (2.7)	0.41 (0.15–1.09)
*P* for trend^c^			0.51		0.42		0.06
*Interferon-γ*							
Q1:>0.57	134 (61.5)	84 (38.5)	1.00	26 (11.9)	1.00	16 (7.3)	1.00
Q2: 0.08–0.57	131 (60.4)	87 (39.6)	0.95 (0.64–1.41)	27 (12.4)	0.82 (0.44–1.53)	13 (6.0)	0.63 (0.28–1.42)
Q3: −0.39–0.07	132 (60.6)	86 (39.4)	0.97 (0.65–1.43)	25 (11.5)	0.84 (0.45–1.56)	11 (5.0)	0.58 (0.26–1.34)
Q4:<−0.39	127 (58.0)	92 (42.0)	1.13 (0.77–1.67)	22 (10.0)	0.84 (0.44–1.58)	9 (4.1)	0.56 (0.23–1.34)
*P* for trend^c^			0.49		0.61		0.18
*Tumour necrosis factor α*							
Q1:>0.87	129 (59.4)	88 (40.6)	1.00	21 (9.7)	1.00	12 (5.5)	1.00
Q2: 0.03–0.87	131 (60.1)	87 (39.9)	0.97 (0.66–1.44)	29 (13.3)	1.39 (0.73–2.62)	15 (6.9)	1.33 (0.58–3.01)
Q3: −0.90–0.02	128 (58.7)	90 (41.3)	1.05 (0.71–1.54)	27 (12.4)	1.38 (0.72–2.61)	12 (5.5)	1.08 (0.46–2.56)
Q4:<−0.90	136 (62.1)	83 (37.9)	0.88 (0.60–1.31)	23 (10.5)	1.01 (0.53–1.96)	10 (4.6)	0.81 (0.33–1.97)
*P* for trend^c^			0.60		0.96		0.56

Abbreviations: CI=confidence interval; OR=odds ratio.

a Change in cytokine values is defined as difference between the geometric mean value of years 1 and 3 and baseline.

b Multivariate OR and 95% CI models were adjusted for age tertiles (<58, 58–66, >66 years), sex, average BMI (<25, 25.0–29.9, ⩾30 kg m^−2^), and current smoking status during the first 3 trial years.

c Median concentrations of each quartile were used to determine *P* for trend for the change in cytokine concentrations.

## Appendix

The members of the Polyp Prevention Study Group participated in the conduct of the Polyp Prevention Trial. However, the data presented in this manuscript and the conclusions drawn from them are solely the responsibility of the above listed co-authors.

*National Cancer Institute*—Schatzkin A, Lanza E, Cross AJ, Corle D, Freedman LS, Clifford C, Tangrea J; *Bowman Gray School of Medicine—*Cooper MR, Paskett E (currently Ohio State University), Quandt S, DeGraffinreid C, Bradham K, Kent L, Self M, Boyles D, West D, Martin L, Taylor N, Dickenson E, Kuhn P, Harmon J, Richardson I, Lee H, Marceau E; *University of New York at Buffalo*—Lance MP (currently University of Arizona), Marshall JR (currently Roswell Park Cancer Center), Hayes D, Phillips J, Petrelli N, Shelton S, Randall E, Blake A, Wodarski L, Deinzer M, Melton R; *Edwards Hines, Jr Hospital, Veterans Administration Medical Center*—Iber FL, Murphy P, Bote EC, Brandt-Whittington L, Haroon N, Kazi N, Moore MA, Orloff SB, Ottosen WJ, Patel M, Rothschild RL, Ryan M, Sullivan JM, Verma A; *Kaiser Foundation Research Institute—*Caan B, Selby JV, Friedman G, Lawson M, Taff G, Snow D, Belfay M, Schoenberger M, Sampel K, Giboney T, Randel M; *Memorial Sloan-Kettering Cancer Center*—Shike M, Winawer S, Bloch A, Mayer J, Morse R, Latkany L, D’Amato D, Schaffer A, Cohen L; *University of Pittsburgh*—Weissfeld J, Schoen R, Schade RR, Kuller L, Gahagan B, Caggiula A, Lucas C, Coyne T, Pappert S, Robinson R, Landis V, Misko S, Search L; *University of Utah*—Burt RW, Slattery M, Viscofsky N, Benson J, Neilson J, McDivitt R, Briley M, Heinrich K, Samowitz W; *Walter Reed Army Medical Center*—Kikendall JW, Mateski DJ, Wong R, Stoute E, Jones-Miskovsky V, Greaser A, Hancock S, Chandler S; *Data and Nutrition Coordinating Center (Westat)*—Cahill J, Hasson M, Daston C, Brewer B, Zimmerman T, Sharbaugh C, O’Brien B, Cranston L, Odaka N, Umbel K, Pinsky J, Price H, Slonim A; *Central Pathologists*—Lewin K (University of California, Los Angeles), Appelman H (University of Michigan); *Laboratories*—Bachorik PS, Lovejoy K (Johns Hopkins University); Sowell A (Centers for Disease Control); *Data and Safety Monitoring Committee*—Greenberg ER (chair) (Dartmouth University); Feldman E (Augusta, Georgia); Garza C (Cornell University); Summers R (University of Iowa); Weiand S (through June 1995) (University of Minnesota); DeMets D (beginning July 1995) (University of Wisconsin).
