# Supplementary material for: Serum cytokine concentrations, flavonol intake and colorectal adenoma recurrence in the Polyp Prevention Trial
Source: Br J Cancer. 2010 Oct 5;103(9):1453–61. doi: 10.1038/sj.bjc.6605915 (PMC2990604; doi:10.1038/sj.bjc.6605915)
Supplement: Supplementary Tables S1–S3 [file 6605915x1.doc]

**Online Supplementary Material**

**Table S1.** Association between the combination of flavonol intake during the trial and change in serum cytokine concentrations from baseline and colorectal adenoma recurrence among intervention group participants of the Polyp Prevention Trial (n = 872)

| **Flavonol**^1^ | **Cytokine**  **Change**^2^ | | | | **Adenoma Recurrence (T4)**^3^ | | | | | | |  |
| --- | --- | --- | --- | --- | --- | --- | --- | --- | --- | --- | --- | --- |
| **(in mg/d)** |  |  |  |  | **No** | **Any** | | **High Risk** | | **Advanced** | |  |
|  | **(in pg/mL)** | | | | ***n* (%)** | ***n* (%)** | **OR (95% CI)**^3^ | ***n* (%)** | **OR (95% CI)**^3^ | ***n* (%)** | **OR (95% CI)**^3^ |  |
| *Mean (T1,2,3)* | *Interleukin (IL)1β* | | | | |  |  |  |  |  |  |  |
| Low ≤ 29.7 | High > 0.01 | | 133 (59.6) | | | 90 (40.4) | 1.00 | 37 (16.6) | 1.00 | 19 ( 8.5) | 1.00 |  |
|  | Low ≤ 0.01 | | 129 (60.6) | | | 84 (39.4) | 0.98 (0.66-1.45) | 25 (11.7) | 0.66 (0.36-1.18) | 17 ( 8.0) | 0.89 (0.43-1.83) |  |
| High > 29.7 | High > 0.01 | | 136 (64.2) | | | 76 (35.8) | 0.99 (0.66-1.49) | 14 ( 6.6) | 0.45 (0.23-0.91) | 8 ( 3.8) | 0.48 (0.19-1.18) |  |
|  | Low ≤ 0.01 | | 126 (56.3) | | | 98 (43.7) | 1.29 (0.87-1.91) | 24 (10.7) | 0.79 (0.43-1.45) | 5 ( 2.2) | 0.31 (0.11-0.88) |  |
| *Mean (T1,2,3)* | | *Interleukin 2* |  | | |  |  |  |  |  |  |  |
| Low ≤ 29.7 | | High > 0.06 | 132 (63.2) | | | 77 (36.8) | 1.00 | 25 (12.0) | 1.00 | 14 ( 6.7) | 1.00 |  |
|  |  | Low ≤ 0.06 | 130 (57.3) | | | 97 (42.7) | 1.28 (0.87-1.90) | 37 (16.3) | 1.46 (0.81-2.62) | 22 ( 9.7) | 1.59 (0.76-3.33) |  |
| High > 29.7 | | High > 0.06 | 142 (62.8) | | | 84 (37.2) | 1.17 (0.77-1.76) | 21 ( 9.3) | 0.93 (0.48-1.82) | 10 ( 4.4) | 0.76 (0.31-1.85) |  |
|  |  | Low ≤ 0.06 | 120 (57.1) | | | 90 (42.9) | 1.49 (0.99-2.25) | 17 ( 8.1) | 0.91 (0.45-1.83) | 3 ( 1.4) | 0.27 (0.07-1.01) |  |
| *Mean (T1,2,3)* | *Interleukin 8* | | |  | |  |  |  |  |  |  |  |
| Low ≤ 29.7 | High > 0.24 | | | 138 (64.8) | | 75 (35.2) | 1.00 | 23 (10.8) | 1.00 | 13 ( 6.1) | 1.00 |  |
|  | Low ≤ 0.24 | | | 124 (55.6) | | 99 (44.4) | 1.39 (0.93-2.05) | 39 (17.5) | 1.76 (0.98-3.18) | 23 (10.3) | 1.78 (0.85-3.73) |  |
| High > 29.7 | High > 0.24 | | | 127 (57.2) | | 95 (42.8) | 1.52 (1.01-2.28) | 18 ( 8.1) | 0.99 (0.49-1.99) | 7 ( 3.2) | 0.60 (0.23-1.62) |  |
|  | Low ≤ 0.24 | | | 135 (63.1) | | 79 (36.9) | 1.21 (0.80-1.84) | 20 ( 9.3) | 1.06 (0.53-2.09) | 6 ( 2.8) | 0.53 (0.19-1.49) |  |
| *Mean (T1,2,3)* | *Interleukin 10* | |  | | |  |  |  |  |  |  |  |
| Low ≤ 29.7 | High > -0.04 | | 140 (59.6) | | | 95 (40.4) | 1.00 | 37 (15.7) | 1.00 | 22 ( 9.4) | 1.00 |  |
|  | Low ≤ -0.04 | | 122 (60.7) | | | 79 (39.3) | 0.95 (0.64-1.41) | 25 (12.4) | 0.70 (0.39-1.26) | 14 ( 7.0) | 0.65 (0.31-1.37) |  |
| High > 29.7 | High > -0.04 | | 122 (61.0) | | | 78 (39.0) | 1.07 (0.71-1.61) | 16 ( 8.0) | 0.57 (0.29-1.13) | 8 ( 4.0) | 0.45 (0.18-1.10) |  |
|  | Low ≤ -0.04 | | 140 (59.3) | | | 96 (40.7) | 1.17 (0.80-1.73) | 22 ( 9.3) | 0.70 (0.38-1.31) | 5 ( 2.1) | 0.26 (0.09-0.72) |  |
| *Mean (T1,2,3)* | *Interleukin 12p70* | | | | |  |  |  |  |  |  |  |
| Low ≤ 29.7 | High > -0.09 | | 132 (60.8) | | | 85 (39.2) | 1.00 | 33 (15.2) | 1.00 | 21 ( 9.7) | 1.00 |  |
|  | Low ≤- 0.09 | | 130 (59.4) | | | 89 (40.6) | 1.05 (0.71-1.55) | 29 (13.2) | 0.83 (0.46-1.47) | 15 ( 6.8) | 0.73 (0.35-1.50) |  |
| High > 29.7 | High > -0.09 | | 131 (60.1) | | | 87 (39.9) | 1.17 (0.78-1.76) | 23 (10.6) | 0.79 (0.42-1.49) | 10 ( 4.6) | 0.53 (0.23-1.25) |  |
|  | Low ≤ -0.09 | | 131 (60.1) | | | 87 (39.9) | 1.19 (0.80-1.79) | 15 ( 6.9) | 0.57 (0.29-1.14) | 3 ( 1.4) | 0.18 (0.05-0.62) |  |
| *Mean (T1,2,3)* | *Granulocyte macrophage colony stimulating factor (GMCSF)* | | | | | | |  |  |  |  |  |
| Low ≤ 29.7 | High > 0.00 | | 141 (59.7) | | | 95 (40.3) | 1.00 | 35 (14.8) | 1.00 | 23 (9.7) | 1.00 |  |
|  | Low ≤ 0.00 | | 121 (60.5) | | | 79 (39.5) | 0.95 (0.64-1.42) | 27 (13.5) | 0.83 (0.46-1.48) | 13 ( 6.5) | 0.62 (0.29-1.31) |  |
| High > 29.7 | High > 0.00 | | 118 (59.3) | | | 81 (40.7) | 1.15 (0.77-1.72) | 17 ( 8.5) | 0.66 (0.34-1.29) | 8 ( 4.0) | 0.46 (0.19-1.11) |  |
|  | Low ≤ 0.00 | | 144 (60.8) | | | 93 (39.2) | 1.11 (0.75-1.64) | 21 ( 8.9) | 0.71 (0.38-1.34) | 5 ( 2.1) | 0.25 (0.09-0.69) |  |
| *Mean (T1,2,3)* | *Interferon (IFN)γ* | | | | |  |  |  |  |  |  |  |
| Low ≤ 29.7 | High > 0.07 | | 137 (60.4) | | | 90 (39.6) | 1.00 | 37 (16.3) | 1.00 | 22 ( 9.7) | 1.00 |  |
|  | Low ≤ 0.07 | | 125 (59.8) | | | 84 (40.2) | 1.05 (0.71-1.55) | 25 (12.0) | 0.75 (0.42-1.34) | 14 ( 6.7) | 0.71 (0.34-1.49) |  |
| High > 29.7 | High > 0.07 | | 128 (61.5) | | | 80 (38.5) | 1.13 (0.75-1.70) | 16 ( 7.7) | 0.56 (0.28-1.11) | 7 ( 3.4) | 0.40 (0.16-1.02) |  |
|  | Low ≤ 0.07 | | 134 (58.8) | | | 94 (41.2) | 1.23 (0.83-1.82) | 22 ( 9.6) | 0.75 (0.40-1.40) | 6 ( 2.6) | 0.32 (0.12-0.85) |  |
| *Mean (T1,2,3)* | *Tumor necrosis factor (TNF)α* | | | | |  |  |  |  |  |  |  |
| Low ≤ 29.7 | High > 0.02 | | 135 (60.0) | | | 90 (40.0) | 1.00 | 33 (14.7) | 1.00 | 19 ( 8.4) | 1.00 |  |
|  | Low ≤ 0.02 | | 127 (60.2) | | | 84 (39.8) | 0.99 (0.67-1.47) | 29 (13.7) | 0.90 (0.50-1.60) | 17 ( 8.1) | 0.91 (0.45-1.88) |  |
| High > 29.7 | High > 0.02 | | 125 (59.5) | | | 85 (40.5) | 1.17 (0.78-1.76) | 17 ( 8.1) | 0.65 (0.33-1.27) | 8 ( 3.8) | 0.50 (0.20-1.24) |  |
|  | Low ≤ 0.02 | | 137 (60.6) | | | 89 (39.4) | 1.13 (0.76-1.68) | 21 (9.3) | 0.78 (0.41-1.47) | 5 ( 2.1) | 0.30 (0.10-0.86) |  |

^1^ Flavonol intake below or above the median of the mean intake during the first 3 trial years (T1,2,3).

^2^ Change in cytokine values is defined as difference between the geometric mean value of years 1 and 3 and baseline.

^3^ Multivariate OR and 95% CI models were adjusted for age tertiles (<58, 58-66, >66 yrs), sex, average BMI (<25, 25.0-29.9, ≥30 kg/m^2^), current smoking status, and average energy intake (continuous) during the first 3 trial years. No significant interaction terms between flavonol intake and change in serum cytokine concentrations were observed for advanced adenoma recurrence. The combination of high flavonol intake and decreased cytokine concentrations had a significantly lower risk estimate of advanced adenoma recurrence for none of the 8 cytokines *vs.* each of the other 3 combinations, for change in IL-1β, IL-12p70, GMCSF, IFNγ, and TNFα *vs.* both lower flavonol intake combinations, for change in IL-2 and IL-8 *vs.* lower flavonol intake and decreased cytokine concentrations, for change in IL-10 *vs.* lower flavonol intake and increased cytokine concentrations, and for none of the 8 cytokines *vs.* high flavonol intake and increased cytokine concentrations.

**Table S2** Association between the combination of flavonol intake during the trial and serum cytokine concentrations at baseline and colorectal adenoma recurrence among intervention group participants of the Polyp Prevention Trial (n = 872)

| **Flavonol**^1^ | **Cytokine**^2^  **(in pg/mL)** | | | **Adenoma Recurrence (T4)**^3^ | | | | | | |  |
| --- | --- | --- | --- | --- | --- | --- | --- | --- | --- | --- | --- |
| **(in mg/d)** |  |  |  | **No** | **Any** | | **High Risk** | | **Advanced** | |  |
|  |  | | | ***n* (%)** | ***n* (%)** | **OR (95% CI)**^‡^ | ***n* (%)** | **OR (95% CI)**^‡^ | ***n* (%)** | **OR (95% CI)**^‡^ |  |
| *Mean (T1,2,3)* | *Interleukin 1β* | |  | |  |  |  |  |  |  |  |
| Low ≤ 29.7 | High > 0.37 | | 130 (58.6) | | 92 (41.4) | 1.00 | 30 (13.5) | 1.00 | 15 ( 6.8) | 1.00 |  |
|  | Low ≤ 0.37 | | 132 (61.7) | | 82 (38.3) | 0.86 (0.58-1.27) | 32 (15.0) | 1.04 (0.58-1.85) | 21 ( 9.8) | 1.31 (0.63-2.71) |  |
| High > 29.7 | High > 0.37 | | 131 (61.2) | | 83 (38.8) | 1.01 (0.68-1.51) | 21 ( 9.8) | 0.83 (0.43-1.58) | 7 ( 3.3) | 0.50 (0.19-1.32) |  |
|  | Low ≤ 0.37 | | 131 (59.0) | | 91 (41.0) | 1.13 (0.76-1.69) | 17 ( 7.7) | 0.70 (0.35-1.39) | 6 ( 2.7) | 0.46 (0.16-1.27) |  |
| *Mean (T1,2,3)* | *Interleukin 2* |  | | |  |  |  |  |  |  |  |
| Low ≤ 29.7 | High > 0.79 | | 128 (59.0) | | 89 (41.0) | 1.00 | 29 (13.4) | 1.00 | 17 ( 7.8) | 1.00 |  |
|  | Low ≤ 0.79 | | 134 (61.2) | | 85 (38.8) | 0.87 (0.58-1.28) | 33 (15.1) | 0.98 (0.55-1.75) | 19 ( 8.7) | 1.00 (0.49-2.07) |  |
| High > 29.7 | High > 0.79 | | 137 (62.8) | | 81 (37.2) | 0.97 (0.65-1.45) | 19 ( 8.7) | 0.71 (0.37-1.38) | 11 ( 5.0) | 0.66 (0.28-1.52) |  |
|  | Low ≤ 0.79 | | 125 (57.3) | | 93 (42.7) | 1.19 (0.80-1.77) | 19 ( 8.7) | 0.78 (0.40-1.52) | 2 ( 0.9) | 0.14 (0.03-0.62) |  |
| *Mean (T1,2,3)* | *Interleukin 8* | |  | |  |  |  |  |  |  |  |
| Low ≤ 29.7 | High > 10.8 | | 139 (61.8) | | 86 (38.2) | 1.00 | 33 (14.7) | 1.00 | 17 ( 7.6) | 1.00 |  |
|  | Low ≤ 10.8 | | 123 (58.3) | | 88 (41.7) | 1.30 (0.87-1.92) | 29 (13.7) | 1.11 (0.62-1.98) | 19 ( 9.0) | 1.36 (0.66-2.80) |  |
| High > 29.7 | High > 10.8 | | 126 (59.7) | | 85 (40.3) | 1.29 (0.86-1.94) | 20 ( 9.5) | 0.84 (0.44-1.60) | 10 ( 4.7) | 0.75 (0.31-1.79) |  |
|  | Low ≤ 10.8 | | 136 (60.4) | | 89 (39.6) | 1.33 (0.89-2.00) | 18 ( 8.0) | 0.74 (0.38-1.45) | 3 ( 1.3) | 0.22 (0.06-0.81) |  |
| *Mean (T1,2,3)* | *Interleukin 10* | |  | |  |  |  |  |  |  |  |
| Low ≤ 29.7 | High > 3.19 | | 131 (60.9) | | 84 (39.1) | 1.00 | 29 (13.5) | 1.00 | 12 ( 5.6) | 1.00 |  |
|  | Low ≤ 3.19 | | 131 (59.3) | | 90 (40.7) | 1.03 (0.69-1.52) | 33 (14.9) | 1.11 (0.62-1.99) | 24 (10.9) | 1.90 (0.89-4.06) |  |
| High > 29.7 | High > 3.19 | | 131 (59.5) | | 89 (40.5) | 1.22 (0.82-1.82) | 21 ( 9.5) | 0.89 (0.47-1.70) | 9 ( 4.1) | 0.85 (0.33-2.16) |  |
|  | Low ≤ 3.19 | | 131 (60.6) | | 85 (39.4) | 1.12 (0.75-1.68) | 17 ( 7.9) | 0.69 (0.35-1.37) | 4 ( 1.9) | 0.36 (0.11-1.18) |  |
| *Mean (T1,2,3)* | *Interleukin 12p70* | | | |  |  |  |  |  |  |  |
| Low ≤ 29.7 | High > 3.01 | | 132 (60.8) | | 85 (39.2) | 1.00 | 26 (12.0) | 1.00 | 13 ( 6.0) | 1.00 |  |
|  | Low ≤ 3.01 | | 130 (59.4) | | 89 (40.6) | 0.99 (0.67-1.47) | 36 (16.4) | 1.37 (0.77-2.45) | 23 (10.5) | 1.65 (0.79-3.46) |  |
| High > 29.7 | High > 3.01 | | 134 (61.5) | | 84 (38.5) | 1.10 (0.74-1.65) | 18 ( 8.3) | 0.85 (0.43-1.68) | 7 ( 3.2) | 0.60 (0.22-1.60) |  |
|  | Low ≤ 3.01 | | 128 (58.7) | | 90 (41.3) | 1.20 (0.80-1.80) | 20 ( 9.2) | 0.93 (0.47-1.83) | 6 ( 2.8) | 0.51 (0.18-1.44) |  |
| *Mean (T1,2,3)* | Granulocyte macrophage colony stimulating factor | | | | | |  |  |  |  |  |
| Low ≤ 29.7 | High > 0.78 | | 131 (60.9) | | 84 (39.1) | 1.00 | 24 (11.2) | 1.00 | 10 ( 4.7) | 1.00 |  |
|  | Low ≤ 0.78 | | 131 (59.3) | | 90 (40.7) | 0.99 (0.67-1.47) | 38 (17.2) | 1.43 (0.80-2.57) | 26 (11.8) | 2.29 (1.04-5.03) |  |
| High > 29.7 | High > 0.78 | | 138 (62.7) | | 82 (37.3) | 1.07 (0.72-1.61) | 16 ( 7.3) | 0.78 (0.39-1.60) | 8 ( 3.6) | 0.85 (0.31-2.30) |  |
|  | Low ≤ 0.78 | | 124 (57.4) | | 92 (42.6) | 1.22 (0.82-1.83) | 22 (10.2) | 1.05 (0.54-2.04) | 5 ( 2.3) | 0.54 (0.17-1.68) |  |
| *Mean (T1,2,3)* | *Interferon γ* | |  | |  |  |  |  |  |  |  |
| Low ≤ 29.7 | High > 1.38 | | 137 (61.2) | | 87 (38.8) | 1.00 | 29 (12.9) | 1.00 | 13 ( 5.8) | 1.00 |  |
|  | Low ≤ 1.38 | | 125 (59.0) | | 87 (41.0) | 1.08 (0.73-1.59) | 33 (15.6) | 1.20 (0.67-2.13) | 23 (10.8) | 1.75 (0.83-3.67) |  |
| High > 29.7 | High > 1.38 | | 122 (57.5) | | 90 (42.5) | 1.31 (0.88-1.96) | 20 ( 9.4) | 0.91 (0.47-1.76) | 9 ( 4.2) | 0.86 (0.34-2.17) |  |
|  | Low ≤ 1.38 | | 140 (62.5) | | 84 (37.5) | 1.08 (0.72-1.63) | 18 ( 8.0) | 0.74 (0.38-1.47) | 4 ( 1.8) | 0.32 (0.10-1.04) |  |
| *Mean (T1,2,3)* | *Tumor necrosis factor α* | | | |  |  |  |  |  |  |  |
| Low ≤ 29.7 | High > 8.18 | | 131 (60.1) | | 87 (39.9) | 1.00 | 29 (12.9) | 1.00 | 16 ( 7.3) | 1.00 |  |
|  | Low ≤ 8.18 | | 131 (60.1) | | 87 (39.9) | 1.21 (0.81-1.79) | 33 (15.6) | 1.35 (0.75-2.43) | 20 ( 9.2) | 1.54 (0.73-3.23) |  |
| High > 29.7 | High > 8.18 | | 135 (61.9) | | 83 (38.1) | 1.10 (0.73-1.65) | 20 ( 9.4) | 1.09 (0.59-2.01) | 9 ( 4.1) | 0.65 (0.27-1.61) |  |
|  | Low ≤ 8.18 | | 127 (58.3) | | 91 (41.7) | 1.47 (0.98-2.23) | 18 ( 8.0) | 0.60 (0.28-1.28) | 4 ( 1.8) | 0.35 (0.11-1.13) |  |

^1^ Flavonol intake below or above the median of the mean intake during the first 3 trial years (T1,2,3).

^2^ Cytokine concentrations below or above the median baseline values (T0).

^3^ Multivariate OR and 95% CI models were adjusted for age tertiles (<58, 58-66, >66 yrs), sex, average BMI (<25, 25.0-29.9, ≥30 kg/m^2^), current smoking status, and average energy intake (continuous) during the first 3 trial years. The interaction term between flavonol intake and serum cytokine concentrations for advanced adenoma recurrence was significant for IL-8, IL-10 and IFNγ. The combination of high flavonol intake and low cytokine concentrations had a significantly lower risk estimate of advanced adenoma recurrence for IL-2 *vs.* each of the other 3 combinations, for IL-8 *vs.* both lower flavonol intake combinations, and for all 8 cytokines *vs.* lower flavonol intake and low baseline cytokine concentrations.

**Table S3** Association between the combination of flavonol intake and serum cytokine concentrations during the trial and colorectal adenoma recurrence among intervention group participants of the Polyp Prevention Trial (n = 872)

| **Flavonol^1^** | **Cytokine**^2^  **(in pg/mL)** | | | **Adenoma Recurrence (T4)** | | | | | | |  |
| --- | --- | --- | --- | --- | --- | --- | --- | --- | --- | --- | --- |
| **(in mg/d)** |  |  |  | **No** | **Any** | | **High Risk** | | **Advanced** | |  |
|  |  | | | ***n* (%)** | ***n* (%)** | **OR (95% CI)**^3^ | ***n* (%)** | **OR (95% CI)**^3^ | ***n* (%)** | **OR (95% CI)**^3^ |  |
| *Mean (T1,2,3)* | *Interleukin (IL)1β* | | | |  |  |  |  |  |  |  |
| Low ≤ 29.7 | High > 0.36 | | 140 (61.1) | | 89 (38.9) | 1.00 | 35 (15.3) | 1.00 | 19 ( 8.3) | 1.00 |  |
|  | Low ≤ 0.36 | | 122 (58.9) | | 85 (41.1) | 1.09 (0.74-1.61) | 27 (13.0) | 0.86 (0.48-1.53) | 17 ( 8.2) | 0.96 (0.47-1.97) |  |
| High > 29.7 | High > 0.36 | | 122 (59.2) | | 84 (40.8) | 1.26 (0.84-1.88) | 23 (11.2) | 0.92 (0.49-1.72) | 9 ( 4.4) | 0.60 (0.25-1.43) |  |
|  | Low ≤ 0.36 | | 140 (60.9) | | 90 (39.1) | 1.15 (0.78-1.71) | 15 ( 6.5) | 0.51 (0.26-1.02) | 4 ( 1.7) | 0.24 (0.08-0.74) |  |
| *Mean (T1,2,3)* | *Interleukin 2* |  | | |  |  |  |  |  |  |  |
| Low ≤ 29.7 | High > 0.71 | | 130 (59.9) | | 87 (40.1) | 1.00 | 32 (14.7) | 1.00 | 17 ( 7.8) | 1.00 |  |
|  | Low ≤ 0.71 | | 132 (60.3) | | 87 (39.7) | 0.97 (0.66-1.44) | 30 (13.7) | 0.92 (0.52-1.64) | 19 ( 8.7) | 1.11 (0.54-2.28) |  |
| High > 29.7 | High > 0.71 | | 136 (62.4) | | 82 (37.6) | 1.05 (0.70-1.58) | 22 (10.1) | 0.86 (0.45-1.62) | 10 ( 4.6) | 0.67 (0.28-1.59) |  |
|  | Low ≤ 0.71 | | 126 (57.8) | | 92 (42.2) | 1.22 (0.82-1.82) | 16 ( 7.3) | 0.59 (0.30-1.17) | 3 ( 1.4) | 0.21 (0.06-0.66) |  |
| *Mean (T1,2,3)* | *Interleukin 8* | |  | |  |  |  |  |  |  |  |
| Low ≤ 29.7 | High > 10.4 | | 132 (59.2) | | 91 (40.8) | 1.00 | 31 (13.9) | 1.00 | 16 ( 7.2) | 1.00 |  |
|  | Low ≤ 10.4 | | 130 (61.0) | | 83 (39.0) | 0.97 (0.66-1.44) | 31 (14.6) | 1.08 (0.61-1.93) | 20 ( 9.4) | 1.30 (0.63-2.69) |  |
| High > 29.7 | High > 10.4 | | 125 (59.0) | | 87 (41.0) | 1.05 (0.70-1.58) | 22 (10.4) | 0.95 (0.50-1.82) | 10 ( 4.7) | 0.78 (0.33-1.89) |  |
|  | Low ≤ 10.4 | | 137 (61.2) | | 87 (38.8) | 1.22 (0.82-1.82) | 16 ( 7.1) | 0.63 (0.31-1.25) | 3 ( 1.3) | 0.21 (0.06-0.76) |  |
| *Mean (T1,2,3)* | *Interleukin 10* | |  | |  |  |  |  |  |  |  |
| Low ≤ 29.7 | High > 3.24 | | 133 (61.5) | | 83 (38.4) | 1.00 | 30 (13.9) | 1.00 | 14 ( 6.5) | 1.00 |  |
|  | Low ≤ 3.24 | | 129 (58.6) | | 91 (41.4) | 1.13 (0.76-1.67) | 32 (14.5) | 1.17 (0.66-2.09) | 22 (10.0) | 1.71 (0.82-3.58) |  |
| High > 29.7 | High > 3.24 | | 132 (60.3) | | 87 (39.7) | 1.22 (0.81-1.83) | 21 ( 9.5) | 0.89 (0.46-1.69) | 10 ( 4.6) | 0.83 (0.34-2.03) |  |
|  | Low ≤ 3.24 | | 130 (59.9) | | 87 (40.1) | 1.23 (0.82-1.84) | 17 ( 7.8) | 0.73 (0.37-1.45) | 3 ( 1.4) | 0.26 (0.07-0.98) |  |
| *Mean (T1,2,3)* | *Interleukin 12p70* | | | |  |  |  |  |  |  |  |
| Low ≤ 29.7 | High > 3.06 | | 136 (63.3) | | 79 (36.7) | 1.00 | 24 (11.2) | 1.00 | 10 ( 4,7) | 1.00 |  |
|  | Low ≤ 3.06 | | 126 (57.0) | | 95 (43.0) | 1.23 (0.83-1.82) | 38 (17.2) | 1.62 (0.90-2.92) | 26 (11.8) | 2.59 (1.18-5.71) |  |
| High > 29.7 | High > 3.06 | | 137 (62.3) | | 83 (37.7) | 1.20 (0.80-1.81) | 18 ( 8.2) | 0.94 (0.47-1.88) | 8 ( 3.6) | 0.92 (0.34-2.49) |  |
|  | Low ≤ 3.06 | | 125 (57.9) | | 91 (42.1) | 1.36 (0.90-2.04) | 20 ( 9.3) | 1.01 (0.51-2.00) | 5 ( 2.3) | 0.57 (0.18-1.80) |  |
| *Mean (T1,2,3)* | *Granulocyte macrophage colony stimulating factor (GMCSF)* | | | | | |  |  |  |  |  |
| Low ≤ 29.7 | High > 0.78 | | 130 (61.6) | | 81 (38.4) | 1.00 | 25 (11.8) | 1.00 | 11 ( 5.2) | 1.00 |  |
|  | Low ≤ 0.78 | | 132 (58.7) | | 93 (41.3) | 1.04 (0.70-1.54) | 37 (16.4) | 1.31 (0.73-2.35) | 25 (11.1) | 1.99 (0.92-4.30) |  |
| High > 29.7 | High > 0.78 | | 139 (62.1) | | 85 (37.9) | 1.11 (0.74-1.67) | 19 ( 8.5) | 0.87 (0.44-1.70) | 10 ( 4.5) | 0.92 (0.37-2.31) |  |
|  | Low ≤ 0.78 | | 123 (58.0) | | 89 (42.0) | 1.24 (0.83-1.87) | 19 ( 9.0) | 0.87 (0.44-1.73) | 3 ( 1.4) | 0.30 (0.08-1.13) |  |
| *Mean (T1,2,3)* | *Interferon (IFN)γ* | | | |  |  |  |  |  |  |  |
| Low ≤ 29.7 | High > 1.30 | | 140 (60.3) | | 92 (39.7) | 1.00 | 37 (15.9) | 1.00 | 20 ( 8.6) | 1.00 |  |
|  | Low ≤ 1.30 | | 122 (59.8) | | 82 (40.2) | 1.00 (0.68-1.48) | 25 (12.3) | 0.69 (0.39-1.25) | 16 ( 7.8) | 0.77 (0.37-1.60) |  |
| High > 29.7 | High > 1.30 | | 124 (60.8) | | 80 (39.2) | 1.12 (0.75-1.67) | 19 ( 9.3) | 0.66 (0.35-1.26) | 11 ( 5.4) | 0.67 (0.30-1.53) |  |
|  | Low ≤ 1.30 | | 138 (59.5) | | 94 (40.5) | 1.19 (0.80-1.77) | 19 ( 8.2) | 0.61 (0.32-1.18) | 2 ( 0.9) | 0.10 (0.02-0.44) |  |
| *Mean (T1,2,3)* | *Tumor necrosis factor (TNF)α* | | | |  |  |  |  |  |  |  |
| Low ≤ 29.7 | High > 8.27 | | 137 (60.1) | | 91 (39.9) | 1.00 | 33 (14.5) | 1.00 | 16 ( 7.0) | 1.00 |  |
|  | Low ≤ 8.27 | | 125 (60.1) | | 83 (39.9) | 1.15 (0.78-1.71) | 29 (13.9) | 1.22 (0.68-2.19) | 20 ( 9.6) | 1.66 (0.80-3.47) |  |
| High > 29.7 | High > 8.27 | | 121 (58.5) | | 86 (41.5) | 1.23 (0.82-1.83) | 21 (10.1) | 0.90 (0.47-1.69) | 9 ( 4.3) | 0.74 (0.30-1.80) |  |
|  | Low ≤ 8.27 | | 141 (61.6) | | 88 (38.4) | 1.24 (0.83-1.86) | 17 ( 7.4) | 0.75 (0.38-1.48) | 4 ( 1.7) | 0.33 (0.10-1.06) |  |

^1^ Flavonol intake below or above the median of the mean intake during the first 3 trial years (T1,2,3).

^2^ Cytokine concentrations below or above the geometric mean values of year 1 and 3 values [Trial (T1,3)].

^3^ Multivariate OR and 95% CI models were adjusted for age tertiles (<58, 58-66, >66 yrs), sex, average BMI (<25, 25.0-29.9, ≥30 kg/m^2^), current smoking status, and average energy intake (continuous) during the first 3 trial years. The interaction term between flavonol intake and serum cytokine concentrations for advanced adenoma recurrence was significant for IL-8, -10, -12p70, and GMCSF. The combination of high flavonol intake and low cytokine concentrations had a significantly lower risk estimate of advanced adenoma recurrence for IFNγ *vs.* each of the other 3 combinations, for IL-1β, -2, -8, and -10 *vs.* both lower flavonol intake combinations, and for all 8 cytokines *vs.* lower flavonol intake and low trial cytokine concentrations.
